# The complete mitochondrial genome of *Sarcophaga angarosinica* (Diptera: Sarcophagidae)

**DOI:** 10.1080/23802359.2023.2233740

**Published:** 2024-02-02

**Authors:** Ziqi Zhou, Zhiyun Pi, Yuanxing Wang, Shaojiang Guo, Nan Guo, Junbo Yang, Xiangyan Zhang, Changquan Zhang, Jifeng Cai

**Affiliations:** a Department of Forensic Science, School of Basic Medical Sciences, Central South University, Changsha, Hunan, China; b Public Security Forensic Center of Haidian, Beijing, China

**Keywords:** Mitogenome, *sarcophaga angarosinica*, sarcophagidae

## Abstract

*Sarcophaga* (*Liosarcophaga*) *angarosinica* (Rohdendorf, 1937) (Diptera: Sarcophagidae) is a species of both medical and ecological significance. In this study, the complete mitochondrial genome (mitogenome) of *S. angarosinica* was sequenced and characterized. The mitogenome has a total length of 15,215 bp, including 13 protein-coding genes, two ribosomal RNAs, 22 transfer RNAs, and an adenine and thymine-rich region. This mitogenome comprises 39.5% adenine, 9.4% guanine, 14.4% cytosine, and 36.8% thymine. Phylogenetic analysis revealed that *S. angarosinica* is closely related to *Sarcophaga similis*. This study enriches the genetic data on *S. angarosinica* and will contribute to establishing the phylogenetic relationships among flesh flies.

## Introduction

In forensic medicine, the sub-discipline of forensic entomology can play an important role in determining the postmortem interval (PMI) (Ren et al. 2018). In this context, the initial piece of evidence is often the identity of the species of insects observed in the vicinity of the corpse, among which dipteran flies tend to be the most commonly encountered. There are approximately 3300 species of flies in the family Sarcophoridae (commonly referred to as flesh flies) worldwide, many of which are associated with carcasses and are characterized by different growth habits. In terms of identification, an analysis of mitochondrial genes is widely used to distinguish fly species, which accordingly necessitates a more complete database of the mitochondrial genomes of sarcosaprophagous insects.

The flesh flies *Sarcophaga angarosinica* (Rohdendorf 1937), which is widely distributed in the central and northern regions of China, is frequently found in association with corpses. However, although this species has forensic entomological significance, its mitochondrial genome has yet to be sequenced. In this paper, we report the first sequencing and characterization of the *S. angarosinica* mitochondrial genome, which will contribute to gaining an understanding of the phylogenetic relationships of *S. angarosinica* and will also be of practical significance from the perspective of forensic entomology.

## Materials

**Figure 1.**
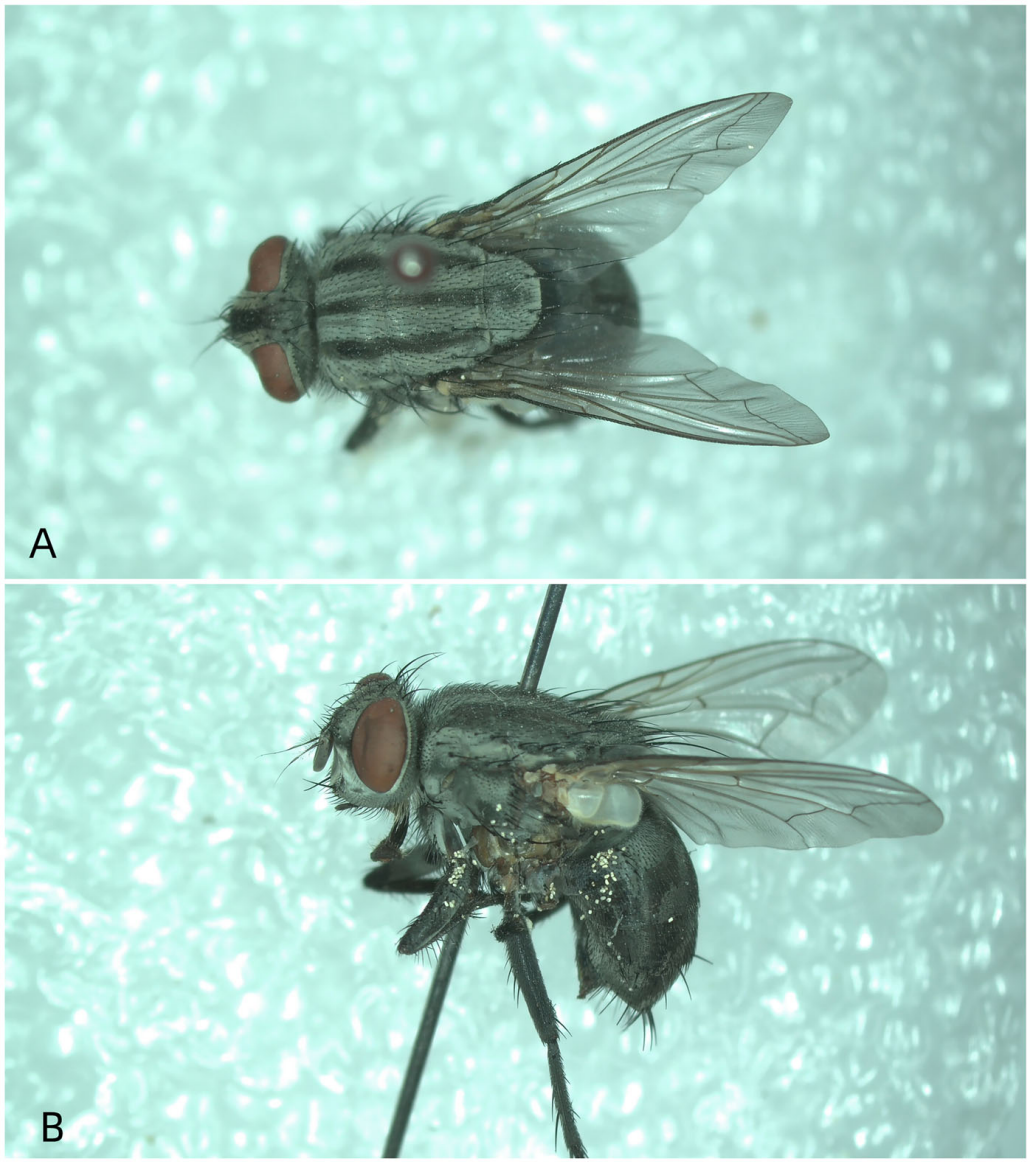
A reference Image of *Sarcophaga angarosinica* sequenced in this work, collected by Xiangyan Zhang in Beijing (40°31′16.53″N, 116°51′54.01″E), China. Photographed by Ziqi Zhou at the Laboratory of Forensic Entomology, Central South University.

The *S. angarosinica* specimens used in this study were obtained from Miyun Reservoir in Beijing in June 2020 (40°31′16.53″N, 116°51′54.01″E) by Zhang Xiangyan from our experimental group (Figure 1). The samples were identified using available traditional morphological keys (Xu and Zhao 1996) and stored at −80 °C for subsequent DNA extraction (Zhang et al. 2019). Test tubes containing each sample were assigned a unique number and have been deposited at the Laboratory of Forensic Entomology, Central South University (location: Changsha, Hunan, China; contact person: Jifeng Cai; email: cjf_jifeng@163.com) under the voucher number (CSU20210203).

## Methods

### Genomic DNA extraction and sequencing

DNA was extracted from the thoracic region of an adult fly using a QIAamp Micro DNA Kit (Qiagen, Hilden, Germany), according to the manufacturer’s protocol. The remaining body parts were preserved for subsequent analysis. The concentration of the extracted DNA was determined using a NanoDrop 2000 spectrophotometer (Thermo Scientific). Thereafter, sequencing was performed using an Illumina NovaSeq 6000 sequencer (PE150). A DNA library with an insert size of 250 base pairs (bp) was constructed using an Illumina® TruSeq® DNA Sample Preparation Kit (Illumina, San Diego, CA, USA) following the manufacturer’s protocol. Paired-end sequencing with a read length of 150 bp (PE 150 bp) was performed using the Illumina HiSeq 2500 platform at OE Biotech Co., Ltd. (Shanghai, China).

### Assembly and annotation

The raw data were trimmed and filtered using Trimmomatic, and de novo mitochondrial assembly was performed using MITObim software. After the rough boundaries of each gene were initially identified using the MITOS2 Web Server. The 13 protein-coding genes (PCGs) within the genome were compared with the published mitoticgenomes of flesh flies using MEGA X (Kumar et al. 2018), and the Open Reading Frame Finder (https://www.ncbi.nlm.nih.gov/orffinder/) was used for further verification.

The Q-INSi method of MAFFT (version 7.263) (Katoh and Standley 2016) was used to separately align the two rRNAs. tRNAscan-SE Search Server (version 1.21) (Lowe and Chan 2016)was used for tRNA prediction and the predicted identities were compared with those of other dipteran insects for verification. The putative A + T-rich region was determined using the Tandem Repeats Finder online server (Benson 1999). Subsequently, circular maps of the mitogenomes were prepared using OGDRAW (version 1.3.1) (Greiner et al. 2019).

### Phylogenetic analysis

For phylogenetic analyses, we used the mitogenome sequences of nine dipteran species in the superfamily Sarcophaginae and the mitogenome sequences of *Calliphora vomitoria* (Diptera: Calliphoridae), which were downloaded from the GenBank database (Shi et al. 2016; Huang and Ma 2018; Yan et al. 2016; Liao et al. 2016; Shang et al. 2019; Ramakodi et al. 2015; Fu et al. 2016; Zhang et al. 2016; Ren et al. 2016,). IQ-TREE (version 1.6.2) (Nguyen et al. 2015) was used to perform maximum likelihood analysis, with nodal support for the majority-rule consensus tree being inferred based on bootstrapping with 10,000 replicates (BPs). Branch support was assessed using bootstrap values (Ren et al. 2020).

## Results

**Figure 2.**
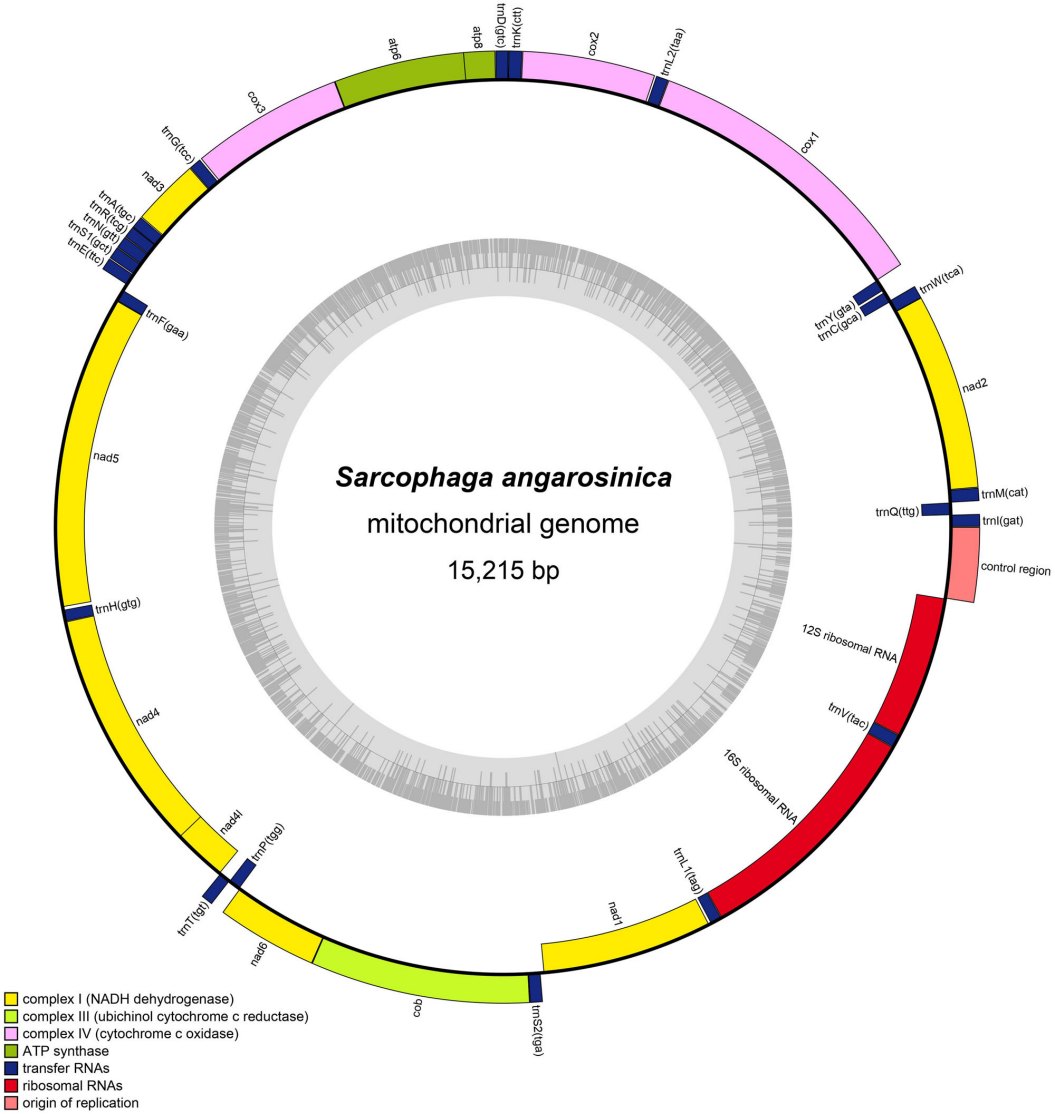
Circular mitogenome map of *S. angarosinica.* The genes scattered on the heavy strand are shown on the outer side of the circle, while the inner side shows those that are scattered on the light strand.

The newly sequenced mitochondrial genomes of *S. angarosinica* have been deposited in the GenBank database (GenBank No. MW592360),. The *S. angarosinica* mitogenome is 15,215 bp in length and consists of 13 protein-coding genes (PCGs), 22 transfer RNAs (tRNAs), two ribosomal RNAs (rRNAs), and a control region (Figure 2). The genome has a base composition of 39.5% adenine, 9.4% guanine, 14.4% cytosine, and 36.8% thymine, and a single A/T-rich region. On the basis of the sequences of the 13 PCGs, all PCGs initiated with a typical start codon of ATN, except that cox1 started with TCG. Most PCGs terminated with TAA/TAG, in addition, four genes (cox1, cox2, nad4, and nad5) terminated with T. Nine PCGs and 14 tRNAs are encoded on the heavy strand (H-strand), and the remaining genes are located on the light strand (L-strand). The genome coverage across the reference figure is detailed in the Supplementary Material (Figure S1).

**Figure 3.**
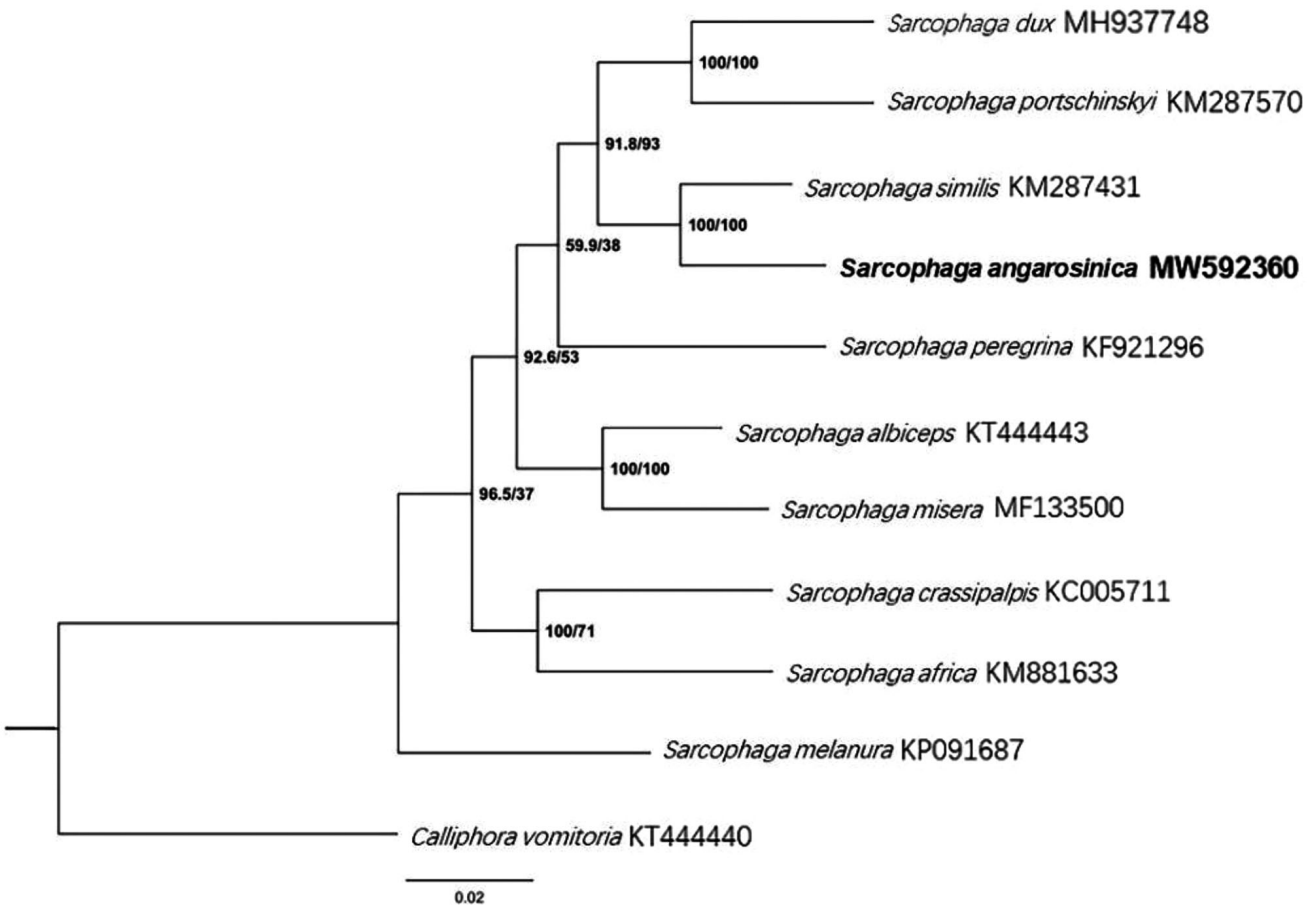
Phylogenetic trees of *S. angarosinica* with nine sarcophagids species based on 13 PCGs by maximum likelihood (ML) method. *Calliphora vomitoria* was selected as an outgroup.

Phylogenetic analysis of *S. angarosinica*, along with a further nine sarcophagid species, was performed using the maximum likelihood method, with *Calliphora vomitoria* (Diptera: Calliphoridae) being used as an outgroup (Figure 3). The phylogenetic tree revealed that *S. angarosinica* is closely related to *Sarcophaga similis* with 100% bootstrap support. However, due to the limited number of specimens, further verification is necessary to confirm whether these species are sister groups.

## Discussion and conclusion

In this study, the mitogenome sequences of *S. angarosinica* are similar in gene number, gene arrangements, and nucleotide composition with the other species in the genus *Sarcophaga*. Gene arrangement of *S. angarosinica* is conserved, like many other species in the Sarcophaginae, retaining the putative ancestral insect mitogenome order (Huang and Ma. 2018). The length of the mitochondrial genome is longer than that of the congeneric reference species, as the result of the variation of length in the A + T rich region.

This study is the first to sequence and characterize the mitochondrial genome of *S. angarosinica*. It enriches the mitochondrial genetic data of *S. angarosinica* to explore the phylogenetic relationships of flesh flies. It also provides basic data support for the practical work of forensic entomology involving *S. angarosinica.*

## Supplementary Material

Supplemental Material

## Data Availability

The data that support the findings of this study are openly available in NCBI at https://www.ncbi.nlm.nih.gov (GenBank: MW592360, BioProject: PRJNA700702, BioSample: SAMN17838722, SRA: SRR13757409).
