# Supplementary figures and images for: The complete mitochondrial genome of Sarcophaga angarosinica (Diptera: Sarcophagidae)
Source: Mitochondrial DNA B Resour. 2024 Feb 2;9(2):242–6. doi: 10.1080/23802359.2023.2233740 (PMC10840587; doi:10.1080/23802359.2023.2233740)

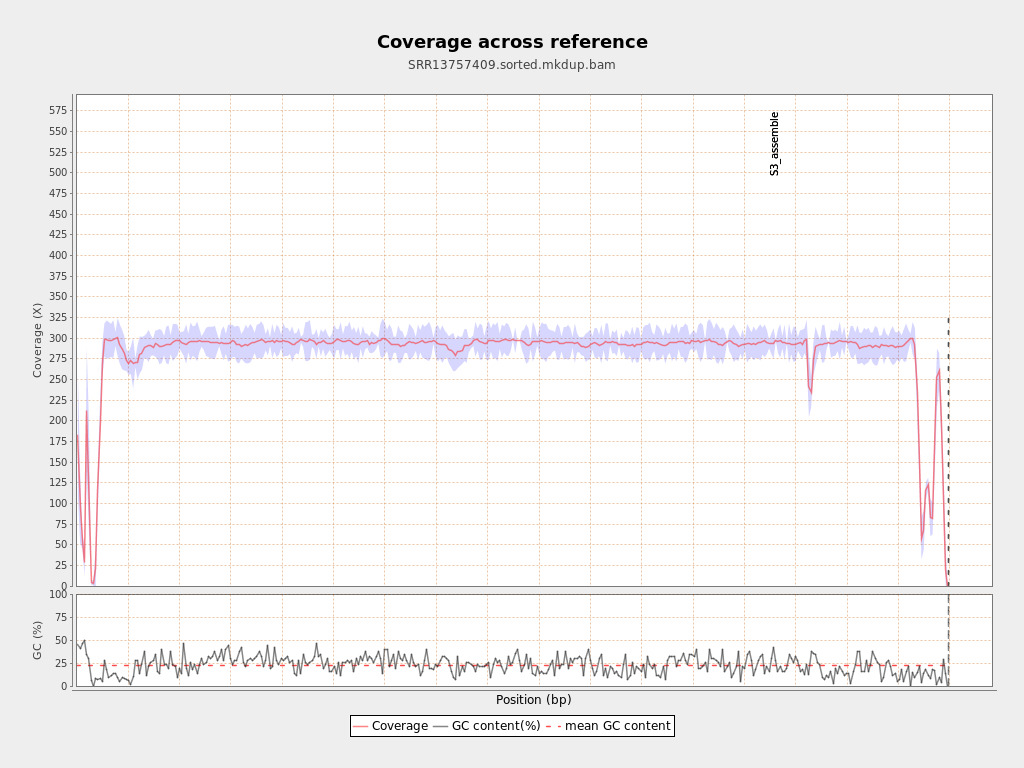

Supplement: Supplemental Material [file TMDN_A_2233740_SM5762.jpg]
